# 8^th^ meeting of the medical assessment consortium UCAN: “Collaborative Perspectives for Competency-based and Quality-assured Medical Assessment”

**DOI:** 10.3205/zma000979

**Published:** 2015-10-15

**Authors:** Ajit Johannes Thamburaj, Konstantin Brass, Manfred Herrmann, Jana Jünger

**Affiliations:** 1 Umbrella Consortium for Assessment Networks, Heidelberg, Deutschland; 2 Universitätsklinikum Heidelberg, Innere Medizin II, Klinik für Allgemeine Innere Medizin und Psychosomatik, Heidelberg, Deutschland; 3 Georg-August Universität, Universitätsmedizin Göttingen, G 1-2 Studiendekanat, Göttingen, Deutschland

## Text

On February 9^th^ and February 10^th^ 2015, the German-speaking members of the “Umbrella Consortium for Assessment Networks (UCAN)” met at the University Medical Center Göttingen for the 8^th^ annual partners meeting. At UCAN, more than 60 institutions from 7 countries work together to create and share new exam contents and formats as well as to support each other in the quality assurance and further development of this content. This year’s meeting focused on *“Collaborative Perspectives for Competency-based and Quality-assured Medical Assessment”*. More than 80 participants from partner institutions as well as external guests shared their experiences about competency-based assessment, collaborative quality assurance and assessment technology in 12 workshops and numerous plenary sessions. In order to effectively deal with the current challenges, (such as the implementation of the National Competency-based Learning target Catalog in Medicine (NKLM) and the recommendations of the German Council for Science and Humanities) the consortium's joint assessment research work shall be intensified with the establishment of an “advisory board for collaborative research”. 

### Shaping tomorrow’s assessment culture through cooperation

**Figure 1:**
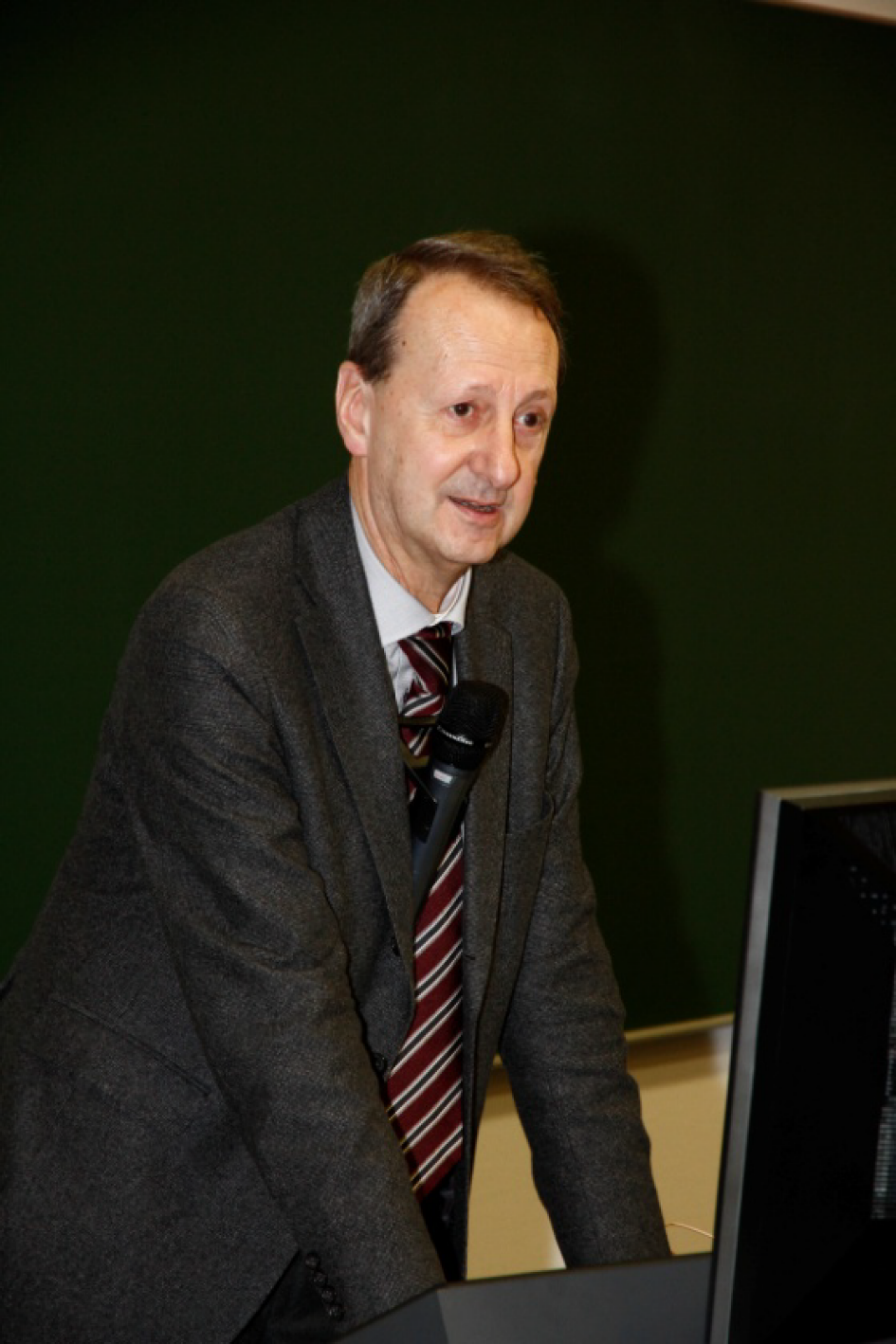
Prof. Dr. Gerhard Burckhardt, study dean at the University Medical Center Göttingen, delivering the keynote speech at the meeting.

“Non scholae, sed vitae discimus” – we learn for life, not for school. With this guiding principle Prof. Dr. Gerhard Burckhardt, Study Dean of the Medical Faculty Göttingen, opened the 8^th^ UCAN partners meeting and reminded the audience that assessment isn’t an end in itself. Highlighting the importance of high quality competency-based exams for a good medical education culture, Burckhardt outlined an agenda as to how students could be enabled to “learn for life” instead of “learning for school” (see figure 1 (Fig. 1)).

Prof. Dr. Burckhardt’s appeal has the finger on the pulse of medical assessment: instead of only testing knowledge, the evaluation of clinical competencies becomes increasingly important. Especially the implementation of the NKLM and the recommendations of the German Council for Science and Humanities are major challenges for the (German) faculties. Consequently, the main question for the 12 workshops was: How can the turn towards competency-based assessment be implemented efficiently without neglecting the quality assurance? 

#### Intensifying collaborative research within the consortium

The workshops tried to find answers for this central question from different perspectives. 

In the workshop *“Designing Shared OSCE Stations”* under the guidance of Dr. Iris Schleicher (Giessen) and Jasmina Sterz (Frankfurt), various partners outlined first standards for the development of OSCE stations that could be shared among the partners.

First exemplary surgery OSCE stations for “appendicitis” and “knee joint” were developed. 

Furthermore, the workshop identified issue areas that need further discussion, such as the standardization of checklist formats and the duration of the stations. 

In the workshop *“Development of a Competency-based Assessment Program for Palliative Medicine”* under the guidance of Dr. Bernd Alt-Epping (Göttingen), Benjamin Ilse (Göttingen), Prof. Dr. Jana Jünger (UCAN) and Stephanie Seidemann (Heidelberg), a competency-based assessment program for palliative medicine was developed, which found the consensus of all attendees. It also became clear that the implementation of such a program can only be achieved together as a network of partners. This would be the best way to tackle most of the currently existing problems like the lack of sufficient working hours dedicated to palliative education or the lack of formats to teach and assess a professional attitude in palliative medicine. In a follow-up session on day two, the group developed first formats for assessing communicative skills in palliative medicine.

**Figure 2:**
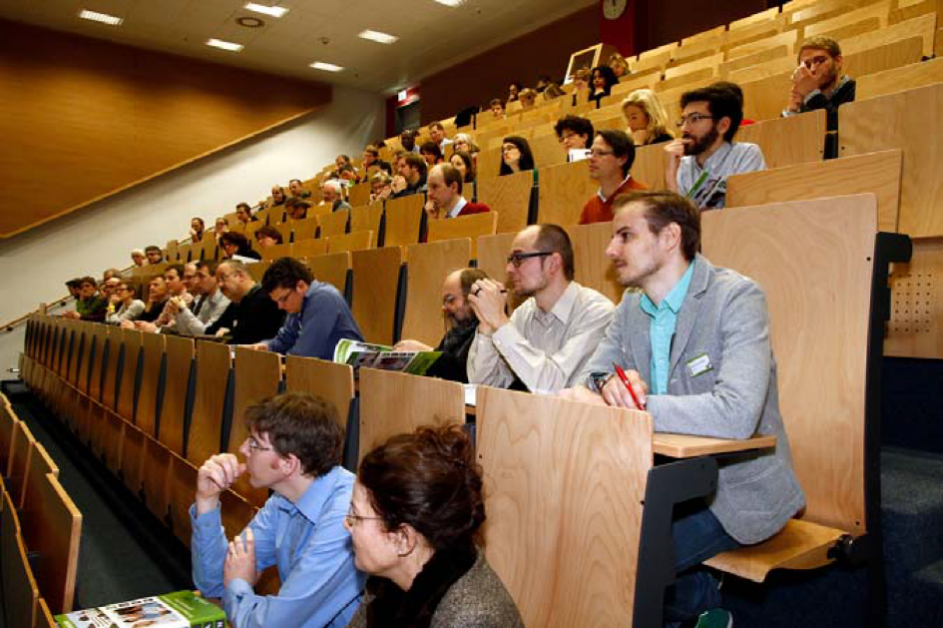
Plenary session at the 8^th^ UCAN partners meeting.

In order to further intensify the joint research in the consortium, an “advisory board for collaborative research” was established at this year’s meeting. The board consists of the assessment coordinators and the designated persons with the qualification of a *Master of Medical Education (MME*) from the UCAN partner faculties (see figure 2 (Fig. 2)). 

#### Further strengthen collaborative quality assurance

During the workshop *“Quality Assurance with the Help of Faculty Reports and the Standardization of Evaluation Methods”* under the guidance of Dr. Andreas Möltner (UCAN) and Dr. Irmgard Streitlein-Böhme (Freiburg), participants discussed how the shared exam platform ItemManagementSystem (IMS) can be utilized for collaborative quality assurance. Besides discussing the existing features, the participants also made suggestions for an easier graphical overview of the test statistics as well as a longitudinal comparison of cohorts with the help of a graphical display. 

In order to further improve the quality of the +200.000 items stored in the database, Dr. Tobias Raupach (Göttingen) conducted a training session titled “Good MC Questions with the Help of the IMS Review and the Blueprinting System”, in which examiners and item authors were trained how to write good MC questions and how the reliability and validity of exams could be enhanced with the IMS quality assurance functions.

One slot was dedicated to an open exchange for the German *Chambers of Physicians (Ärztekammern)* organized in UCAN. One of the main topics was how the participating chambers could use the integrated IMS review system for their quality assurance requirements. 

In the workshop *“Competency-based Student Progress Test”* under the guidance of Stefan Wagener (Heidelberg) and Felicitas Eckrich (UCAN), students had the chance to give feedback on the recently conducted progress test and how the student feedback after the test could be improved. The Student Progress Test is a format in which specially trained students create and review the exam content themselves. To generate test questions, “subject groups” (based on the medical licensure act, ÄAppO) and “competency areas” (based on the NKLM) were combined to create a two-dimensional blueprint. 

#### Large interest in tablet-based exams

In various technical workshops, the UCAN Partners could learn more about the latest technical developments and new features of the UCAN Tools. During one session, participants learned how to efficiently use the new features of the *Examinator* for running their post-exam evaluation and test statistics as well as how to use the integrated scanner tool “*EDGAR”*. In another session, the new features of the IMS were presented, especially how items can be authored with the completely redesigned item editor. Dr. Manfred Herrmann from the University Medical Center Göttingen also gave a presentation on how they plan, prepare and conduct desktop computer-based electronic exams with the UCAN Tool “Campus”. During these sessions, the developers also got important feedback from the users as to how the system could be further improved. 

One of the most-frequented events of this year's conference was the workshop “*Conducting Exams on Tablet Computers”* under the guidance of Jörn Heid (UCAN). Apart from the new features of the well-established tOSCE app for tablet-based OSCEs, the UCAN developer team also presented the new app “tEXAM”: with this app, Partners can deliver MCQ exams created with the IMS on tablet computers. Various partners gave a presentation on their first experiences with this new app. 

**Figure 3:**
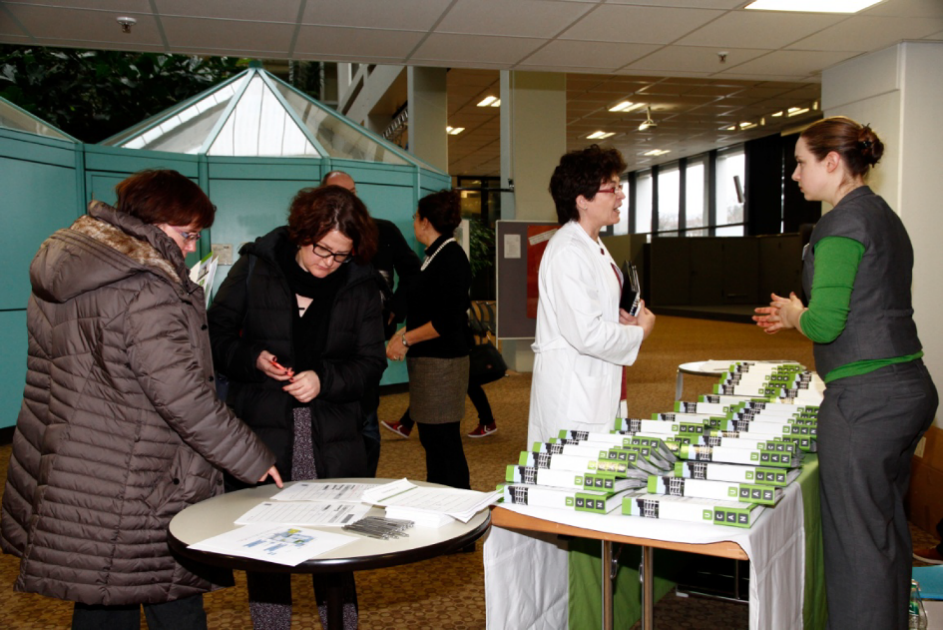
Registration desk in the lounge of the University Medical Center Göttingen.

In addition to the 15 partners that already use tOSCE, many more partners have announced that they want to switch from paper-based checklists to tOSCE for their practical exams (see figure 3 (Fig. 3)). 

#### 6 new partners join UCAN

In the plenary, six new partners that have recently joined UCAN gave a short presentation about their assessment programs:

1. The Medical Faculty of the University of Halle is now a full UCAN partner and uses the ItemManagementSystem for various exams at their institution.
2. With the Karl Landsteiner University of Health Sciences, a second institution from Austria joined the network.
3. The medical specialist society *Foederatio Chirurgicorum Helvetica (fmch)* joined UCAN in 2014 and uses the IMS for its Basisexamen Chirurgie.
4. The Swiss Society Oto-Rhino-Laryngology (ORL) also joined UCAN last year and uses the IMS for its MCQ exam.
5. The Schweizer Gesellschaft für Intensivmedizin (SGI) conducts its joint exam with the European Society for Intensive Care Medicine (ESICM) with the help of UCAN Tools. The societies use the IMS, tOSCE and the Examinator for an exam held simultaneously in eight different European cities.
6. With the Touchtstone Institute form Toronto (Canada), UCAN also welcomes its first Partner from North America. The biggest medical assessment center of its kind in Canada assesses internationally educated health professionals (nurses, doctors, optometrists) for their readiness for the residency programs in the state of Ontario.

#### 10^th^ anniversary meeting next year in Heidelberg

The partners meeting concluded with a final plenary session, in which the main results of the conference were summarized. The final documentation of the conference can be obtained from the UCAN office on request.

Next year's conference will be the 10^th^ anniversary of UCAN and is going to be held in Heidelberg. More information on the meeting as well as on the medical assessment consortium UCAN can be found at www.ucan-assess.org.

## Competing interests

The authors declare that they have no competing interests.

